# Need for a precise molecular diagnosis in Beckwith-Wiedemann and Silver-Russell syndrome: what has to be considered and why it is important

**DOI:** 10.1007/s00109-020-01966-z

**Published:** 2020-08-24

**Authors:** Thomas Eggermann, Johanna Brück, Cordula Knopp, György Fekete, Christian Kratz, Velibor Tasic, Ingo Kurth, Miriam Elbracht, Katja Eggermann, Matthias Begemann

**Affiliations:** 1 Institute of Human Genetics, Medical Faculty, RWTH Aachen University, Pauwelsstr. 30, D-52074 Aachen, Germany; 2 II. Department of Pediatrics, Semmelweis University, Budapest, Hungary; 3 Department of Pediatric Hematology and Oncology, Hannover Medical School, Hannover, Germany; 4 University Children’s Hospital, Skopje, Macedonia

**Keywords:** Silver-Russell syndrome, Beckwith-Wiedemann syndrome, MS MLPA, Diagnostics, Detection rate

## Abstract

**Abstract:**

Molecular diagnostic testing of the 11p15.5-associated imprinting disorders Silver-Russell and Beckwith-Wiedemann syndrome (SRS, BWS) is challenging due to the broad spectrum of molecular defects and their mosaic occurrence. Additionally, the decision on the molecular testing algorithm is hindered by their clinical heterogeneity. However, the precise identification of the type of defect is often a prerequisite for the clinical management and genetic counselling. Four major molecular alterations (epimutations, uniparental disomies, copy number variants, single nucleotide variants) have been identified, but their frequencies vary between SRS and BWS. Due to their molecular aetiology, epimutations in both disorders as well as upd(11)pat in BWS are particular prone to mosaicism which might additionally complicate the interpretation of testing results. We report on our experience of molecular analysis in a total cohort of 1448 patients referred for diagnostic testing of BWS and SRS, comprising a dataset from 737 new patients and from 711 cases from a recent study. Though the majority of positively tested patients showed the expected molecular results, we identified a considerable number of clinically unexpected molecular alterations as well as not yet reported changes and discrepant mosaic distributions. Additionally, the rate of multilocus imprinting disturbances among the patients with epimutations and uniparental diploidies could be further specified. Altogether, these cases show that comprehensive testing strategies have to be applied in diagnostic testing of SRS and BWS. The precise molecular diagnosis is required as the basis for a targeted management (e.g. ECG (electrocardiogram) and tumour surveillance in BWS, growth treatment in SRS). The molecular diagnosis furthermore provides the basis for genetic counselling. However, it has to be considered that recurrence risk calculation is determined by the phenotypic consequences of each molecular alteration and mechanism by which the alteration arose.

**Key messages:**

- The detection rates for the typical molecular defects of Beckwith-Wiedemann syndrome or Silver-Russell syndrome (BWS, SRS) are lower in routine cohorts than in clinically well-characterised ones.
- A broad spectrum of (unexpected) molecular alterations in both disorders can be identified.
- Multilocus imprinting disturbances (MLID) are less frequent in SRS than expected.
- The frequency of MLID and uniparental diploidy in BWS is confirmed.
- Mosaicism is a diagnostic challenge in BWS and SRS.
- The precise determination of the molecular defects affecting is the basis for a targeted clinical management and genetic counselling.

## Introduction

**Fig. 1:**
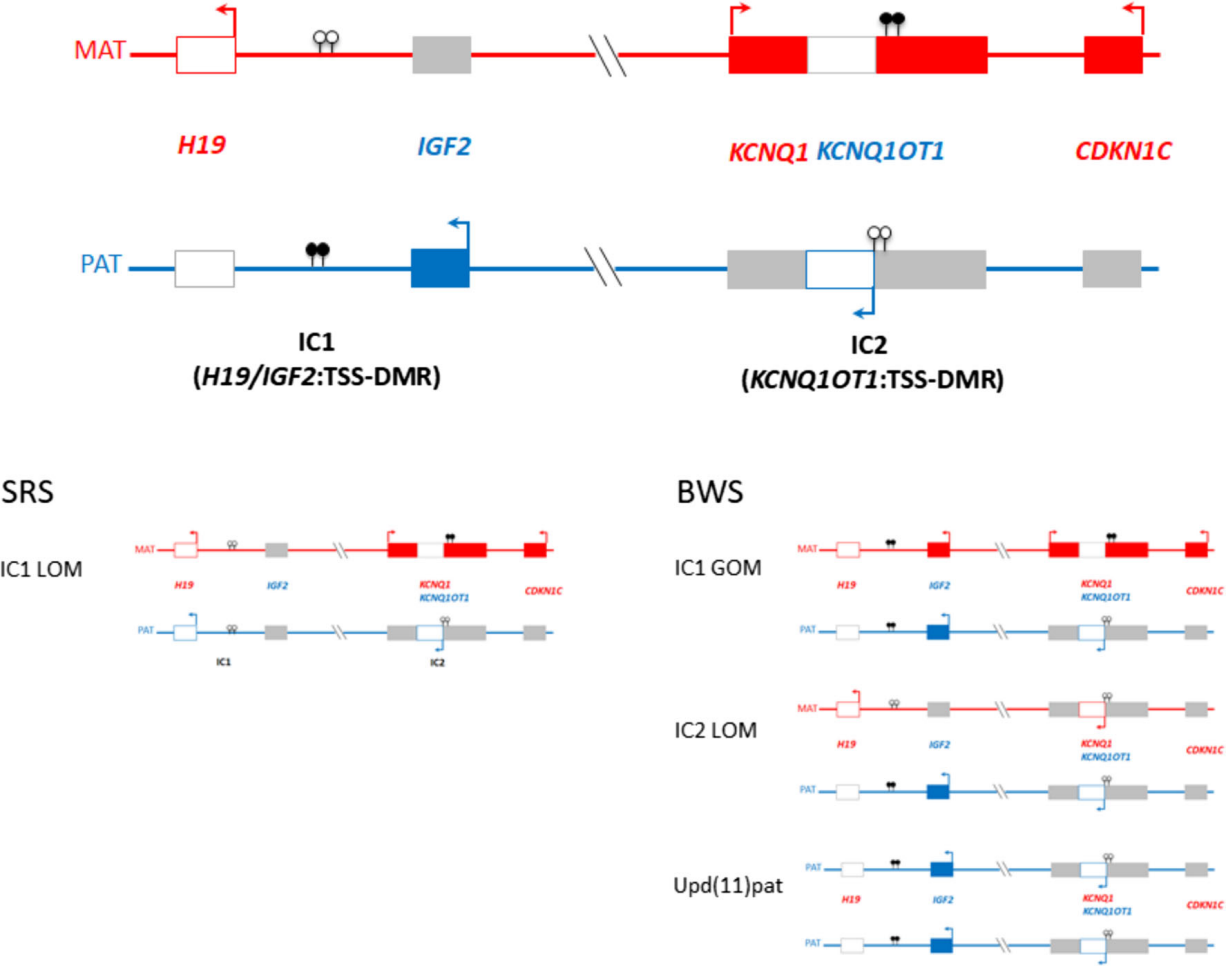
The two imprinting domains in the chromosomal region 11p15.5 and the major disturbances which might occur in SRS and BWS. (In red: genes expressed from the maternal allele, in blue: gene expressed from the paternal allele, full boxes: protein-coding genes, empty boxes: non-coding RNAs; lollipops: DMRs, filled lollipops: methylated DMR, empty lollipops: unmethylated DMR)

The chromosomal region 11p15.5 harbours two differentially methylated regions (DMRs), the Imprinting Centre regions 1 and 2 (IC1, IC2) (Fig. 1). The IC1 (IC1, *H19/IGF2*:TSS-DMR) is paternally methylated and regulates the expression of the maternally expressed *H19* gene and the paternally expressed *IGF2* gene. In contrast, the IC2 (*KCNQ1OT1*:TSS-DMR) is maternally methylated, resulting in the expression of the *KCNQ1OT1* gene from the paternal and of *CDKN1C* from the maternal allele. Disturbances of both regions alter human growth and are associated with two imprinting disorders, Beckwith-Wiedemann and Silver-Russell syndromes (BWS, OMIM130650; SRS, OMIM180860). The characteristic feature of BWS is overgrowth; additionally, patients often exhibit body asymmetry, macroglossia, and umbilical wall defects [1]. For clinical management, the increased risk to develop (embryonal) tumours has to be considered, though this risk depends on the molecular defect in 11p15.5. In contrast to BWS, SRS is characterised by severe intrauterine and postnatal growth retardation, associated with relative macrocephaly, a typical triangular face due to a protruding forehead and a pointed chin, asymmetry, and feeding difficulties [2]. For both syndromes, the precise determination of the disease-causing molecular defect is crucial for clinical management, in particular as BWS, and some differential diagnoses of SRS are tumour predisposition syndromes [1, 3].

The opposite growth features of BWS and SRS are reflected by opposite molecular alterations in 11p15.5 (Fig. 1). The molecular spectrum of BWS mainly comprises loss of methylation (LOM) of IC2 in nearly 50% of patients, paternal uniparental disomy of 11p15.5 (upd(11p15)pat, ~ 20%), gain of methylation (GOM) of IC1 (5–10%), copy number variants (CNVs) affecting 11p15.5 (< 5%), and pathogenic single nucleotide variants (SNVs) in the *CDKN1C* gene (~ 5% of sporadic, up to 50% of familial cases)(for review: [1]). In total, these alterations account for nearly 80% of patients with a phenotype indicative for BWS [1], leaving a diagnostic gap of nearly 20%. In SRS, LOM of the IC1 is the major disturbance (40–60% of patients), whereas other 11p15.5 alterations are rather rare. Up to 10% of patients diagnosed as SRS carry a maternal UPD of chromosome 7 (upd(7)mat) [2]. Molecular changes restricted to 14q32 and consistent with Temple syndrome (TS14, OMIM616222) are increasingly reported, due to the clinical overlap between these imprinting disorders [4, 5]. Finally, submicroscopic CNVs can be observed as well [2, 6]. In addition, for clinical and molecular diagnosis of both disorders, differential diagnoses have to be considered [1, 2]. A common observation in patients with epimutations in both disorders, i.e. patients with LOM or GOM, and BWS patients with upd(11p15)pat is the mosaic occurrence of the disturbances, which represents a challenge for diagnostic detection. As molecular genetic testing is commonly restricted only to one tissue (e.g. lymphocytes from peripheral blood), epimutations and upd(11)pat might escape detection due to the highly discrepant mosaic distribution with an apparent normal methylation pattern in the investigated cell system and an abnormal methylation in another (e.g. [7, 8]). In the LOM groups of BWS and SRS, as well as in LOM patients from other imprinting disorders, a significant number of patients exhibit multilocus imprinting disturbances (MLID), i.e. in these patients more imprinted loci than the disease-specific one are affected by hypomethylation (for review: [9]). In several MLID families, the so-called pathogenic variants in maternal effect genes have been identified which mediate the proper imprint marking in the oocyte [10].

With the growing knowledge on the molecular spectrum and the availability of commercially available diagnostic assays, genetic testing for the 11p15.5-associated imprinting disorders has been expanded. In particular, methylation-specific multiplex ligation-dependent probe amplification (MS MLPA) has meanwhile been well-established and is applied in many diagnostic laboratories [11]. However, the decision to refer a patient for genetic testing of SRS or BWS is often hindered by the breadth of the clinical spectrum, the non-specificity of symptoms (particular in SRS), and the overlap with other congenital disorders. To circumvent this problem and to standardise the clinical diagnosis, clinical scoring systems for both disorders have been suggested [1, 2], and in cases with typical features/high clinical scores, the detection rates for the different molecular subtypes mentioned above can be achieved. However, the general detection rates in unselected populations referred for molecular testing of SRS and BWS are lower, because clinical scoring is either not possible due to the lack of clinical data, or has not been conducted, or the referring clinicians aim to exclude the molecular alterations associated with the two diseases. On the other hand, clinicians do not want to miss a molecular diagnosis of BWS (and SRS) due to the possible consequences for the clinical management of the patients and genetic counselling. As a result, the diagnostic yield in patients referred for routine diagnostic testing is much lower than that in clinically well-characterised cohorts [12]. However, testing of patients not exhibiting the full clinical pictures of SRS and BWS shows that the clinical spectrum is very broad, and in particular, in SRS, several molecular SRS patients have been identified who do not fulfil the clinical diagnostic criteria. The inclusion of further imprinted loci in genetic testing therefore allows for the identification of rare molecular alterations.

In order to further estimate the detection rate for the common molecular changes in BWS and SRS in a routine diagnostic cohort, and to get an overview on rare and/or neglected (epi)mutations, we analysed the results from or diagnostic cohort ascertained from January 2014 to February 2020. These data were compiled with a dataset from a previous study [12]. Based on our data, we overview the molecular spectrum in this cohort, and we aim to make the readers aware of the complexity and the consequences of genetic testing in this heterogeneous group of disorders.

## Study cohort and methods

The cohort consisted of 502 patients referred for molecular diagnostic testing for SRS, and 235 patients with clinical features indicating BWS between January 2014 and February 2020 to the Institute of Human Genetics in Aachen, Germany. Additionally, prenatal testing was performed in 5 pregnancies with ultrasound findings indicating SRS (i.e. intrauterine growth retardation) and 39 pregnancies with BWS features. Signed informed consents were obtained from all patients and/or their care holders. Some of the positive testing results had already been published (Table 1). To interpret the data on a broader number of cases, the data from this new cohort were compiled with a dataset from a previous study [12]. The study was approved by the ethical committee of the Medical Faculty, RWTH Aachen University (EK303-18).

**Table 1:** Summary of the molecular findings obtained by MS MLPA approaches in routine diagnostic cohorts of patients referred for SRS and BWS testing. Data from the cohorts ascertained from 2014 to 2020 (new data) are compiled with those from a previous study [12]. (Publications on specific molecular alterations and cases are indicated: a [13], b [10], c [14], d [15], e [16], f [17], g [18]) (total numbers are given in parentheses)

Molecular finding	SRS			BWS		
	This study	Previous study	Total (%)	This study	Previous study	Total (%)
IC1 LOM	69.8% (74)	68.4% (78)	69.1	2.9% (3a)		2.1
IC1 GOM				12.6% (13)	5.0% (2)	10.5
IC2 LOM	2.8% (3a)		1.4	37.9% (39)	45.0.0% (20)	41.3
MLID	1.8% (2b)	5.3% (6)	3.6	11.7% (12b)	15% (5)	11.9
upd(11)pat				28.2% (29)	22.5% (9)	26.6
uniparental diploidy				1.9% (2c)	5.0% (2)	2.8
upd(7)mat	14.1% (15)	17.5% (20)	15.9			
upd(6)mat	0.9% (1d)	0.9% (1)	0.9			
14q32 alterations	6.6% (7)	1.8% (2)	4.1			
11p15.5 CNVs	2.8% (3e)	4.4% (5)	3.6	4.9% (5f)	5.0% (2)	4.9
chromosome 7 alterations	0.9% (1g)	1.8% (2)	1.4			
Total number of aberrant findings	106	114	220	103	40	143
Total number of tests	502	571	1073	235	140	375
Detection rate	21.0%	19.9%	20.5	43.8%	28.6%	38.1

Genomic DNA was isolated from peripheral lymphocytes; in nearly thirty cases, additional tissues were available. All samples were tested by methylation-specific multiplex ligation-dependent probe amplification with the assays ME030 targeting the IC1 and IC2 in 11p15.5 (MRC Holland, Amsterdam, The Netherlands). In case of SRS, the assay ME032 with further probes for the imprinted loci on chromosomes 6, 7, and 14 was used as well. The results of all patients were routinely confirmed by the ME034 assay targeting imprinted loci on chromosomes 6, 7, 11, 14, 15, 19, and 20 (*PLAGL1*-DMR (6q24), *GRB10*:alt-TSS-DMR (7p12), *MEST*:alt-TSS-DMR (7q32), *H19/IGF2*:IG-DMR (11p15), *KCNQ1OT1*:TSS-DMR (11p15), *MEG3*:TSS-DMR (14q32), *SNURF*:TSS-DMR (15q11), *PEG3*:TSS-DMR(19q13.43), *GNAS-NESP*:TSS-DMR (20q13), *GNAS-AS1*:TSS-DMR (20q13), *GNAS-XL*:Ex1-DMR (20q13)). By this approach, MLID affecting the clinically relevant imprinted loci were identified in a one-tube reaction. MLID was diagnosed in case at least one imprinted locus in addition to the disease-specific DMR was affected. Additionally, upd(11)pat could thereby be discriminated from uniparental diploidy. In case a further molecular characterisation was needed, microsatellite typing to confirm UPD or molecular karyotyping (CytoScan™ HD, Affymetrix, Santa Clara/CA, USA) was conducted. Mutation analysis of the coding region of the *CDKN1C* gene (NM_000076.2) was performed by Sanger sequencing; primers are available on request.

## Results

In the group of 502 newly ascertained children referred for SRS testing, molecular alterations detectable by MS MLPA were identified in 21.0% (*n* = 106) (Table 1).

The majority consisted of IC1 LOM (69.8%); in two of them, MLID was present. Among the 14.1% of upd(7)mat carriers, one had a segmental upd(7q)mat (arr[hg19] 7q11.23q36.3(76550200-159119220) hmz). The 14q32 alteration subgroup (6.6%) comprised three carriers of a upd(14)mat, three patients with epimutations, and one patient in which the discrimination between upd(14)mat and epimutation was not possible.

An LOM of the IC2 which is typically associated with a BWS phenotype was observed in three cases referred for SRS testing (2.8%). CNVs of or within 11p15.5 was identified in another three cases (2.8%); they comprised a deletion of the paternal *KCNQ1OT1* copy, and two gains of maternal 11p15.5 material affecting both IC1 and IC2. Two further rare findings (upd(6)mat) and a 7q32 deletion affecting the *MEST* gene were detected as well.

Out of the 235 newly ascertained patients for which BWS testing was requested, 43.8% of patients (*n* = 103) exhibited a molecular alteration at least in 11p15.5. With 37.9%, LOM of the IC2 was the most frequent finding; additionally, 11.7% of patients with IC2 LOM were found to have MLID. upd(11)pat accounted for 28.2% of patients; in two further cases, a uniparental diploidy was present. IC1 GOM occurred in 12.6% of patients, but in three cases, IC1 LOM corresponding to a molecular diagnosis of SRS was identified. CNVs of or within 11p15.5 were present in 4.9%. The latter comprised a deletion in the IC1 on the maternal allele, and three duplications affecting the paternal IC1 (and IC2) copies.

Testing of additional tissues confirmed the clinical diagnosis in one SRS patient with the typical phenotype (5 out of 5 parameters of the Netchine-Harbison clinical scoring system for SRS [19]). He carried a IC1 LOM in DNA from finger nails, whereas lymphocyte testing had given a negative result. Lymphocyte testing was also negative in two patients referred as BWS, but in nephroblastoma, a IC1 GOM and a upd(11p15)pat could be identified, respectively.

Prenatal testing for SRS was requested only for 5 pregnancies due to intrauterine growth restriction, and all tests were negative. In the prenatal BWS cohort, two out of 39 samples were positively tested (one IC2 LOM, one upd(11p15)pat). The reason for prenatal testing in these two cases was abnormal ultrasound findings consistent with BWS; further details were not provided.

**Fig. 2:**
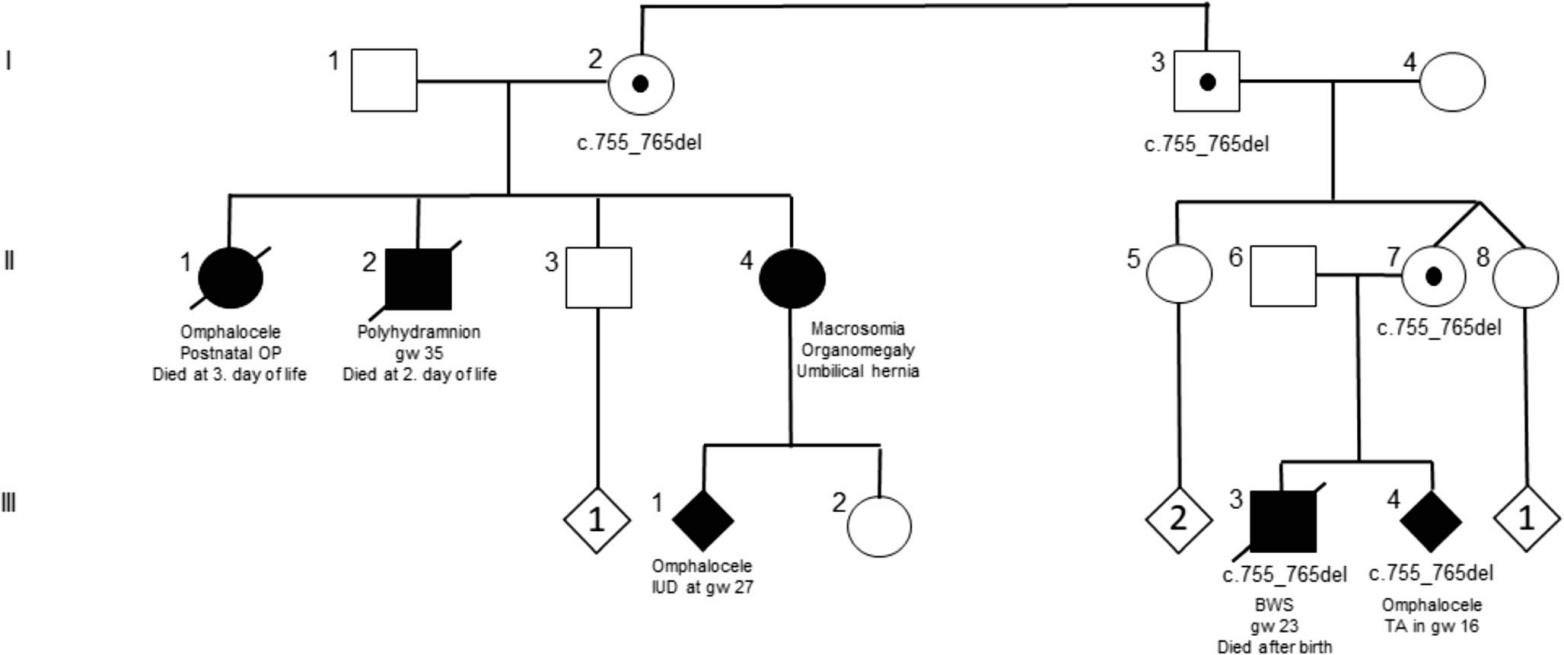
Pedigree of the family with an 11-bp deletion in *CDKN1C* (NM_000076.2:c.755_765del (Chr11(GRCh38): g.2884725_2884735del; p.(Ala252Glyfs*30)). According to the inheritance of *CDKN1C* variants, clinical features only occur in case of maternal inheritance of the variant. However, the family is unusual because of the severity of the *CDKN1C*-associated phenotypes. (OP operation; IUD intrauterine death, TA therapeutic abortion)

In 36 patients, *CDKN1C* testing was requested after exclusion of the major 11p15.5 alterations, among them were three prenatal cases. After a negative MS MLPA result, a pathogenic variant was identified in a male foetus with a large omphalocele who died during the delivery after premature rupture of the membranes in gestational week (gw) 23 (Fig. 2). The foetus (III.3) inherited a heterozygous 11-bp deletion from his healthy mother (II.7) (NM_000076.2:c.755_765del (Chr11(GRCh38): g.2884725_2884735del; p.(Ala252Glyfs*30))). This frameshift variant has not yet been reported in public databases (dbSNP, gnomAD). It is localised in the PCNA (proliferating cell nuclear antigen) binding domain which binds CDKN1C to PCNA and thereby affects DNA replication and repair. The loss-of-function variant NM_000076.2:c.755_765del thus affects CDKN1C as a negative regulator of cell proliferation and might thereby cause an overgrowth phenotype. In a second pregnancy, the foetus (III.4) was heterozygous for the variant as well and showed an omphalocele. The heterozygous grandfather (I.3) had a sister (I.2) who gave birth to three children with anomalies that could be classified as BWS features retrospectively (II.1, II.2, II.4). A foetus of II.4 was affected by i.a. omphalocele, and pregnancy was terminated in gw 27.

## Discussion

With the improved availability of MS MLPA as molecular test for SRS and BWS, the number of test orders and the number of laboratories offering these tests are growing. As a consequence, genetic testing for both disorders is increasingly required by clinicians even in case only single clinical signs are observed, and the intention is often rather to exclude the frequent molecular findings of BWS and SRS than to confirm them. As a result, the detection rates for the typical molecular defects are lower than in clinically well-characterised cohorts [7, 12]. On the other hand, an increasing number of different molecular alterations in both disorders is reported, and by the combined application of different MS MLPA assays, this spectrum can at least in part be covered. By compiling our data from diagnostic testing for SRS and BWS from the last years (this study and those from our previous dataset [12]), we overview the spectrum of molecular defects affecting clinically relevant imprinted loci in both disorders which are the basis for a more precise clinical management and genetic counselling.

The comparison of new diagnostic data on SRS testing obtained from 2014 to January 2020 with those from our previous study [12] confirmed our overall diagnostic yield of approximately 20%. In BWS, the detection rate had increased from 28.6 to 43.8% which might be explained in part by the different sizes of the cohorts and the improved awareness of the referring clinicians for the clinical features of the syndrome.

For SRS, the frequencies of the molecular subgroups could be further established. Whereas the frequency of IC1 LOM and upd(7)mat as the major alterations is similar in both cohorts (about 69% and 16%, respectively); it becomes obvious that 14q32 alterations affecting the *MEG3*:TSS-DMR account for a significant number of cases (5%). This frequency of 14q32 disturbances among patients referred for SRS testing could be expected as there is a growing number of reports on the clinical overlap of TS14 and SRS at least in the neonate and early childhood period (e.g. [4, 5]). We therefore suggest to include the 14q32 locus in the first trial of molecular SRS testing. As SRS is a clinical diagnosis [2], patients with molecular TS14 might be regarded as another molecular subgroup, and accordingly, it has to be discussed whether TS14 is indeed an owned entity [5].

In the laboratory workup, the inclusion of the *MEG3*:TSS-DMR is easily possible as this DMR is already included in the commercially available ME032 assay which also addresses upd(7)mat. The advantage of the use of (multilocus) MS MLPA assays is also obvious from the additional findings we obtained in our cohort (Table 1). The spectrum of molecular disturbances in growth-retarded patients referred for SRS testing also includes upd(6)mat, and chromosome 7 alterations other than upd(7)mat. The latter help to narrow down the chromosome 7 region which might harbour genes involved in the aetiology of SRS [18]. They comprise CNVs in 7q32 (14), as well as patients in which segmental uniparental disomies are restricted to parts of the long arm of chromosome 7 (upd(7q)mat). In our total cohort of 51 cases with upd(7) ascertained in the last two decades, we identified four upd(7q)mat cases. It should be noted that in all cases, the region 7q32 with the imprinted *MEST* locus is affected; therefore, this region represents a conclusive candidate gene region. However, as single SRS description with structural variants of 7p13 (including *GRB10* [20]) show, this region should also be analysed.

In BWS, the frequencies of the molecular subgroups corresponded to that of previous studies (e.g. [1, 21, 22]), with IC2 LOM as the most frequent findings, followed by upd(11)pat. In 9.5% of cases diagnosed as upd(11)pat (4 out of 42), a mosaic paternal uniparental diploidy (also known as genome-wide uniparental disomy) could be identified. Patients with upd(11)pat generally have the highest risk to develop embryonic tumours within the first years of life among BWS patients [1]. However, carriers of paternal uniparental disomy nearly always develop a broad range of tumours, associated with higher mortality and a life-long cancer risk [14, 23].

MLID is a shared subgroup among SRS and BWS patients, and it is characterised by an aberrant imprinting of loci in addition to the disease-specific epimutations, LOM of IC1 and IC2, respectively. Whereas the frequency of approximately 25% of MLID among the IC2 LOM patients with BWS phenotype confirms the data from other studies [24, 25]; the rate of MLID among our SRS is lower than initially expected and reported [24]. However, the different studies conducted to identify MLID are not standardised in respect to the targeted imprinted loci and methods. Furthermore, MLID occurs as mosaic, and therefore, it might escape detection [8]. The patterns of aberrant methylation in MLID patients referred with BWS or SRS phenotypes was obviously not different [10, 26], though the 14q32 DMRs were apparently more often affected in patients with SRS than with BWS phenotypes (Brück: personal communication).

As recent data about the aetiology of MLID show, these patients should be identified as their mothers might carry the so-called maternal effect variants which make them prone for reproductive failures. These maternal effect variants account for up to 50% of MLID cases [10] and affect proteins of the subcortical complex of the oocyte [9] which mediates the proper imprinting in embryonic development. Women carrying maternal effect, variants are healthy, but are at a higher risk for children with disturbed imprinting without predictable phenotypes, hydatidiform moles, aneuploidies, and miscarriages [27, 28]. In case maternal effect variants can be identified, an adapted reproductive management including oocyte donation should be discussed [29]. The detection of MLID might be challenging due to the variable degree of mosaicism in different tissues AZZI and the lack of a clinical correlate. However, we suggest to generally consider MLID testing in the routine diagnostic strategy of BWS and SRS since reliable MS MLPA assays are commercially available. At least in families with more than one child with an epimutation and/or a history of reproductive failure (miscarriages, hydatidiform moles, aneuploidies), MLID is strongly suggested.

In addition to the broad spectrum of 11p15.5 alterations, the detection of molecular findings which are not expected from the clinical reason for referral is another challenge in the diagnostics of SRS and BWS. As we could show with our new data, both IC2 LOM can be identified in patients referred for SRS testing, and vice versa IC1 LOM for BWS. In fact, different reasons for these at first glance, unexpected findings have been suggested [13]; among them are mosaic distributions of the epimutation, ambiguous and unspecific clinical features, and phenotypic overlaps.

In addition to epimutations and uniparental disomy, CNVs also contribute to a significant number of cases (up to 5% in BWS) (Table 1). In general, the clinical outcome of 11p15.5 CNVs depend on the parental origin of the affected allele, but the size and genetic content of the variant also has an impact on the phenotype. Duplications affecting both IC1 and IC2 are a major group among 11p15.5 CNVs, and they either cause a BWS phenotype in case of gain of paternal genetic material, or SRS features if the maternal allele is affected. However, the situation becomes more diverse in case only one of the ICs or even smaller elements within the ICs are affected (for review: [30]). Here the phenotype depends on the function of the affected segment, particularly if recognition and binding sites for transcription factors are affected (for review: [31]). Additionally, these structural variants might be caused by familial chromosomal rearrangements (e.g. [32]). In summary, in case of CNVs, further molecular and cytogenetic analyses have to be performed to characterise the variant and their possible familial transmission, as the basis for a precise counselling and prediction.

In case of UPD detection, analysis referring to familial inheritance is not indicated in case of upd(11p)pat as this mutation nearly always occur de novo, and also for upd(7)mat, familial transmittance is extremely rare and should be discussed only in case of a family history indicating a chromosomal disturbance (e.g. miscarriages, several affected family members) [33].

The situation is different in case of pathogenic SNVs in imprinted genes in SRS and BWS. In fact, the only gene in which a significant number of variants have been identified in 11p15.5-associated imprinting disorders is *CDKN1C*. Variants in this gene account for nearly 5% of patients with BWS, and it is even higher in familial cases, as confirmed in our family with a CD*KN1C* frameshift variant (Fig. 2). However, *CDKN1C* variants are rare in SRS, as are variants in *IGF2* as the second gene for which SNVs have been reported in single SRS families [2]. Therefore, routine testing for variants in these two genes is not indicated in the first diagnostic trial which should be methylation-specific testing [1, 2], but both genes should be included in next-generation sequencing–based approaches as the next step of molecular SRS and BWS diagnostics. The clinical findings in the patients from our family corroborate the observation that frameshift variants are associated with more severe phenotypes, including omphalocele [34]. However, the clinical course in the affected family members is extraordinarily severe, and the reason is currently unclear.

A challenge in the molecular diagnosis of SRS and BWS is mosaicism which has to be anticipated in case of epimutations (IC1 GOM, IC1 LOM, IC2 LOM) and upd(11p15)pat. The mosaic distribution of these alterations can be very tissue-specific; therefore, an inconspicuous testing result in one tissue does not exclude epimutation or upd(11p15)pat in another cell system. To circumvent these false-negative results, the use of different methods and analyses of different tissues might be discussed [7, 8]. However, the chance to identify the disease-causing alteration in another tissue has to be estimated in conjunction with the clinical plausibility, the burden for the patient and the family, the costs, and efforts. We suggest to restrict the analysis of further tissues to those cases fulfilling clinical criteria for SRS or BWS, based on the criteria consented recently [1, 2].

The precise molecular diagnosis is required as the basis for a targeted management of SRS and BWS patients. For SRS, major questions in respect to therapy and prognosis refer to growth hormone treatment, pubertal development, feeding, and neurodevelopment [2, 35–37]. Currently, only limited data are available on the efficiency of growth hormone treatment in the different molecular subgroups, but it is has to be taken into account that some of the differential diagnosis of SRS are tumour predisposition syndromes [3]. Motor and speech delays are common in SRS (for review: [2]), but a more global developmental delay can be observed in upd(7)mat patients, requiring an adequate management. In addition, particular carriers of CNVs might be prone to developmental delay depending on the gene content and size of the chromosomal variant (for review: [6]).

Tumour monitoring is in the focus of clinical management BWS, and a correlation between molecular subgroups and tumour risks is obvious (for review: [1]). However, we strongly recommend to test patients with initial diagnosis of upd(11)pat for a paternal uniparental diploidy (“genome-wide UPD”) due to the significantly high risk to develop a broad range of tumours during their life (see also: [1]). Additionally, in patients with IC2 LOM, testing of MLID should be considered. Though it is currently unclear whether MLID patients exhibit a different phenotype than “pure” IC2 LOM carriers, at least for genetic counselling, the discrimination might become relevant due to maternal effect variants (see above).

In general, the precise definition of the molecular subtype is required for specific genetic counselling. The identification of a monogenetic cause (e.g. maternal effect variants, pathogenic variants in *CDKN1C*, and further differential diagnosis genes) helps to delineate the recurrence risk. Familial-inherited CNVs might predispose to different clinical pictures, depending on the nature of the CNV, its gene content, and the sex of the transmitting parent in case of imprinted regions. Finally, the history of the family of SRS and BWS patients should be carefully documented, as congenital dysmorphisms and malformations as well as reproductive failures might help to identify the molecular cause of the disease.

In summary, the molecular results from a large diagnostic cohort of patients referred for SRS and BWS testing and tested by multilocus MS MLPA illustrate the broad range of alterations which are associated with similar clinical phenotypes. Though some of these disturbances are rare, they present the basis of an adapted therapy and counselling, and can easily be detected by the same testing approach. The application of new high-throughput (methylation-specific) techniques (e.g. next-generation sequencing–based) will increase the specificity and sensitivity of diagnostic tests and thereby further improve the diagnostic yield and accuracy of the assays. However, the growing number of different molecular alterations resulting in similar phenotypes (e.g. SRS and TS14) puts the definition of the different disorders as clinical entities in questions and requires a discussion on their redefinition.

## Data Availability

Data are available on request.
